# Dual-Space Information Modeling of Socio-Economic Systems under Information Asymmetry

**DOI:** 10.3390/e21050528

**Published:** 2019-05-24

**Authors:** Lei Bao, Joseph Fritchman

**Affiliations:** The Ohio State University, Columbus, OH 43210, USA

**Keywords:** information, agent based modeling, complex systems, information asymmetry, computational economics

## Abstract

Information definitions across many disciplines commonly treat information as a physical world entity. Information measures are used along with other physical variables undistinguished for modeling physical systems. Building on previous work, this research explicitly defines information as a unique category of entity that is created by intelligent agents to represent aspects of the physical world but that is not part of the physical world. This leads to the formation of the dual-space information modeling (DSIM) framework, which clearly distinguishes an information space from the physically based material space. The separation of information and material spaces allows new insight and flexibility into modeling complex systems. In this research, DSIM based agent models are applied to study the impact of information asymmetry to marketing behaviors. This paper demonstrates the effectiveness of the DSIM framework in the modeling process and how emergent behavior from these systems is encapsulated in the dual-space.

## 1. Introduction

Information dominates much of today’s society and plays a prominent role in modern technology and the lives of everyday people. Information is often broadly used as the knowledge or representation about aspects of the physical world. However, the very nature of information is still loosely defined and varies across different disciplines and classes of problems [1,2,3]. The fundamental question on whether information is part of the physical nature or whether it is an intelligence-based entity remains unanswered. Among the many discipline-based and philosophical definitions, information is mostly understood as being a part of the physical world to describe the characteristics (or values) of the output of a process and its input [3]. The current literature suggests that information is predominately treated as an entity of the physical world.

In a recent study, this view of information as an entity of the physical world has been challenged with a dual-space information modeling (DSIM) framework that explicitly separates an information space from the physical material space [4]. In this framework, the material space includes the entire physical world consisting of material entities and interactions. The information space, on the other hand, exists solely within intelligent agents, which have their physical forms as part of the material space but can encode descriptive or representational patterns to describe behaviors and processes in both material and information spaces. These representation patterns are created and maintained by the intelligent agents and form the basis of information in the DSIM framework.

Although all information is encoded using specifically conditioned material space phenomena, the information itself can be “external” to the material space behaviors that the information is encoded with and can represent features of other material or information processes [4]. The concept of information being external to material entities is a key defining concept that leads to the dual-space structure in the DSIM framework. Overall, the information space itself can contain multiple broad categories of information including: (1) local information that describes a system’s internal/local level properties, (2) external information that is encoded based on a system’s states but is used for representing entities that are external to the system, and (3) system-state information that provides statistical descriptions of the system. These conceptual definitions help clarify differences between the material properties of a system and its information entities. However, it is left to an intelligent agent to define, develop, and utilize this information.

A fundamental issue with the treatment of information as merely an output entity of a physical process is that this makes information dependent on the original physical process and implies that the ontology of information is a physical world entity as a product of a physical process and that it represents features of the very physical process that creates the information. As a result, the view that the essence of information can be external to a physical system or process, which means that information that is encoded within certain physical entities is used to represent features of other physical or informational entities, is not comprehensible from the perspective that treats information as part of the physical world. That is, the discretion and definition of using certain physical phenomena to encode information to represent features of other physical systems or information entities can only be determined and conducted by intelligent agents.

In the DSIM framework, information is an intelligence-based representation of material and information processes and is created and maintained by intelligent agents. The output of a physical process could be encoded by an intelligent agent and reproduced and shared among other intelligent agents, which are independent of the original physical process. That is, information can evolve within the intelligent agent community without the original physical system and process leading to the creation of the information.

This paper seeks to clarify the nature of information, from what it is to how it interacts with the physical world, as well as how it is created and transmitted or shared. Attention will be given to distinguishing information from the actual physical world. In the following sections, the DSIM framework will be reviewed and applied to create dual-space agent and information models (AIM) to study market behaviors under varying information asymmetry.

## 2. The Dual-Space Information Model

Researchers in many fields have found it beneficial to consider the effects of information on their systems of interest. However, many still treat information as a physical reality within their systems. Recently it has been proposed that there are benefits to more carefully defining and separating the physical world and information into two distinct spaces: the material (or physical) space and the information space, which is referred to as the dual-space information modeling (DSIM) framework [4]. As discussed earlier, the core concept leading to the DSIM framework is the idea that the property of information is external to a physical system with which the information is encoded. This feature is a direct consequence of the probabilistic representation of information. The definitions of both a probabilistic system and the corresponding encoding framework can be arbitrary and are determined by intelligent agents.

In most works within the current information theory, stable states of a physical system are used as symbols by intelligent agents to encode information that is used for external purposes, such as in applications of digital storage and communication [5,6,7]. These states of a physical system exist in a probability distribution. To define this distribution, the entire system and its possible states need to be known and form a normalized complete set that can be used to define the probability of the occurrence of a specific state. Operationally, a system is always defined by an intelligent agent (an observer) that can include an arbitrary set of entities from the material space. The very concept of a system is an information entity defined by intelligent agents and does not exist in the material space. Therefore, a system and its probabilistic states are both defined by an intelligent observer as information entities, which are fundamentally different from and external to the material entities included in the defined physical system. This difference provides the conceptual underpinning for separating the information and material spaces.

The encoding of information using the probabilistic states of a system has been discussed in detail in a previous study [4]. Basically, any physical system that has distinct stable states can be used to encode information. If a system is truly random, the probability of any state occurring is uniform; however, when a system is conditioned with artificial rules or physical laws, certain states will be much more likely to occur. To quantify information, Shannon and Gibbs define the scale of information that can be encoded in a state as the inverse of the probability of a specific state occurring: $h_{i}=-\log\left(p_{i}\right)$ [5]. States which occur naturally (frequently at equilibrium) are then only able to encode a minimal amount of information. However, states that have low probabilities of naturally occurring would be able to encode a higher degree of information.

Current information theory focuses mostly on the technical side of using a system’s states as symbols for encoding information but does not address the nature of information. The encoding properties of a system, such as the capacity and uncertainty, are based on the system’s distribution of probability states, which are defined by intelligent observers and are external to the system. The system’s states act merely as the medium to encode, store, and transmit information. This is most obvious with the category of “external information”, in which the encoded information does not describe the internal properties of the system’s constituents but is used to represent entities external to the system [4].

For example, the state of a digital circuit system can have multiple patterns of 0 s and 1 s. Such patterns can be encoded as information by an intelligent agent (an external entity to the circuit) to represent features of another physical system. The actual circuit system only maintains and changes the 0–1 states based on its physical interactions and has no interpretation of the information or meaning encoded within the 0–1 patterns. Such information is only meaningful to the external intelligent agent who defines the information. In this circuit example, the content of the information is also external to the circuit system that the information is encoded with. However, there can be cases in which the externally defined information is also used to represent the features of the physical system that the information is encoded with. With the circuit example, the encoded information can also be used to represent certain internal states of the circuit itself, in which case the content of the information becomes internal, but the information is still externally defined by an intelligent agent.

**Figure 1:**
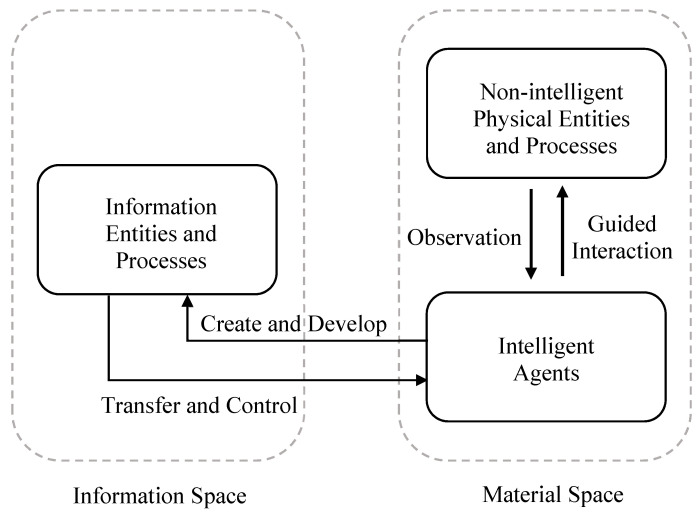
The components and processes of the Dual-Space Information Modeling framework.

To summarize, in the DSIM framework, the nature of information is explicitly defined as an intelligent-based representation of material and information space entities. Information is created, maintained, transferred, applied, and evolved solely within the community of intelligent agents. Intelligent agents are defined as physical entities that can accomplish some or all of the information tasks. These can exist in many forms of biological, mechanical (e.g., artificial intelligence), or mixed bio-mechanical constructs at wide ranging levels of complexity. The representation of information requires a definition of a system and its probability structure, which can only be defined by an intelligent agent, and the concept of a system is external to the physical entities involved within the defined system. Figure 1 provides a simple schematic diagram to illustrate the components, relations, and processes of the DSIM framework.

As illustrated in Figure 1, none-intelligent physical entities in the material space simply exist and interact in reality and do not have the intelligent constructs to create information even about their own existence. When the physical entities (and their interactions) are observed by an intelligent agent, information is created by the intelligent agent to represent and describe the features of the physical entities either purposefully or automatically. Information can also be continuously developed and shared among intelligent agents without the involvement of the actual physical systems represented by the information. The interactions between information and physical entities have to go through intelligent agents, which are material space entities who also create and develop information entities. Through intelligent agents, information can evolve and develop in its own space and can also make tangible impacts to the material space.

The DSIM framework can be implemented with the agent-based modeling approach to create agent-information models (AIM) to study and analyze complex systems [4]. The components of the dual spaces and their interactions are context dependent and need to be defined for the systems that are being modelled. As part of any analysis of complex systems, it is useful to generally understand the interactions among components of the dual spaces, which allow four types of interactions:

1. Material-material: These are physical interactions among entities in the material space (such as collision and motion under forces), which require no information to explain how two objects interact in the absence of intelligence.
2. Information-information: These are interactions among information entities carried out by intelligent agents. Typical interactions include using existing information to make a decision and or create new information. The information involved and produced are not physical realities and exist only in the information space maintained by the intelligent agents.
3. Material-information: These are interactions carried out by intelligent agents, in which intelligence agents make observations of material space entities and create information to describe aspects of the material space entities.
4. Information-material: These are interactions carried out by intelligent agents, in which intelligent agents apply information in order to physically impact material space entities.

Among the four types of interactions, three involve information and have to be conducted by intelligent agents using their intelligent functionalities. The first type is within material space only and does not involve any intelligence. It is worth noting that the physical body of an intelligent agent is a material space entity and is always involved in physical-only interactions with other material space entities, such as water going through human cells to support biological functions, which are medial-material interactions in view of their physical realities. As long as the intelligent function is not engaged, information will not be part of such interactions.

Computationally, the different types of interactions can be expressed as a recursive system of equations (Equation (1)) [4]. For a system at time t, the material space is represented by M(t), and the information space is represented by I(t):

(1) $$\begin{array}{c} M\left(t+\Delta t\right)=F\left(M\left(t\right),I\left(t\right)\right)\\ I\left(t+\Delta t\right)=f\left(M\left(t\right),\;I\left(t\right)\right) \end{array}$$

Here, F and f are system dependent functions, which define how information is created and develops and how information influences material interactions. Implicitly, F and f depend on the whole history of each space. The concept of an intelligent agent is embedded within the system functions and expressed as agent functions in AIM models.

In this research, the DSIM framework will be applied to study a socio-economic example of a complex system, observing marketing behavior under varying levels of information asymmetry. Economic models have studied information for decades, leading to several landmark studies. For example, the 2001 Nobel prize in economics was awarded to George Akerlof, Michael Spence, and Joseph E. Stiglitz “for their analyses of markets with asymmetric information” [8], the 2005 Nobel prize was awarded to Robert J. Aumann and Thomas C. Schelling, “for having enhanced our understanding of conflict and cooperation through game theory analysis” [9], and recently, the 2017 prize was awarded to Richard Thaler, “for his contributions to behavioral economics” [10]. Each of these (and many other studies) have utilized versions of information to aid in the study of economic decision making. The simplest use of information in decision-making is a single condition if-then process (if the price is below some amount, buy; if it is above that amount, sell). In most studies, information is used primarily to define the rules of decision-making processes (e.g., set the price an individual will buy or sell at, or establish a set of rules individuals will follow), in which the focus is placed on the decisions made and consequences of these decisions. Meanwhile, the dynamics of information itself are directly determined by the material space entities and do not have their own evolution [4].

Asymmetric information deals with differences in the information that two individuals have obtained (e.g., the actual vs. the perceived value of an object), and how they make decisions with the available information. The prime example of this is Akerlof’s “The Market for Lemons” [11]. Spence proposed the idea of signaling in order to aid in resolving the asymmetry (e.g., earning a college degree can function as a signal of the level of an individual’s ability to learn) [12]. Stiglitz added the theory of screening, whereby the underinformed individual may induce the other to reveal their information [13]. While information can be exchanged, the focus is primarily on the perceived value of material. Each of these uses information in a way that directly decides what action will be taken—information translates directly as conditions leading to a decision—making outcome.

Furthermore, game theory develops models of conflict and cooperation between intelligent decision makers. Much of the research in economics focuses on finding solutions or equilibria, which tend to fit these models in a box that either describes how populations behave or suggests how individuals should behave. Evolutionary game theory focuses on the dynamics of strategy changes. Within stable systems, these typically focus on finding the final equilibrium state of a system. For example, Aumann’s Nobel contribution applied fundamental equilibrium concepts of noncooperative game theory (perfect, strong, and correlated equilibria) to repeated noncooperative games [14]. A cooperative outcome ensures that no player can guarantee a better outcome for themselves, though it may not be achievable in equilibrium. However, the repetition of games acts as a reinforcement that leads to the emergence of cooperative outcomes in equilibrium (a player taking a greedy approach now may lead to others punishing that player later).

Information in the context of game theory typically refers to the set of all possible moves that have or could have taken place in the game so far, based on the observations of a player. Based on the available information, and desired outcomes, a player will plan their next move accordingly. In Aumann’s repeated games, information acts as an error correction feedback for players to choose strategies focusing on long-term benefits rather than short-term gains. This leads to a focus on the resulting equilibrium state. In these cases, information is used as a criterial condition in decision-making to choose what action to take based on a predefined algorithm.

In addition, behavioral economics studies the effects of psychological, social, and cognitive factors on economic decision-making and the consequences of actions taken. Thaler’s work established that people are predictably irrational in ways that defy economic theory through three phases of reactions to information: underreaction, adjustment, and overreaction [15,16].

The types of information used in behavioral economics are quite varied but tend to focus on the perceived values involved in a trade and what nudges will elicit a reaction. A common example for perceived value is that people generally will not pay more for an umbrella while it is raining than they would otherwise. Their perceived value of the umbrella does not change despite changes in usefulness. An example of a simple nudge used is that the British government added pictures of vehicles to billing letters for car registration fees, which made people more likely to pay. However, both uses of information here (and indeed many in general) focus on the physical results of the information changes and not the creation and evolution of the information itself. In fact, here information or beliefs are used to choose between sets of predefined actions to be taken. The types of information biases and actions that people would take have already been pre-determined (known) through other cognitive and psychological studies. The economic models simply select such results for use to achieve desired actions.

The examples above all have in common that they use information or beliefs to develop a concrete set of rules for the system to follow. These rules are typically unchanging throughout a model (e.g., an agent will seek to maximize its performance over the course of a simulation). Functionally, this results in resorting to a series of if-then statements (if specific conditions exist, then agents will take actions with some certainty). Factoring in an agent’s beliefs alters the conditions that are necessary for these if-then rules. Still, the focus of most research is on the results of sets of beliefs on the system, and not on the underlying intelligent processes which may evolve information independently of the material space. That is, the information itself is not designed to allow an independent evolution [4]. There are very limited studies that use information in more intelligent ways so that information is allowed to evolve separately from the material space. For example, Pastore, Ponta, and Cincotti [17,18,19] studied markets that use information as sentiments about the market, which would determine how much of an asset an agent would buy or sell. While the sentiments reflected a perceived valuation of an asset, they were free to evolve based on an individual’s view of the market and influences spread from connected agents.

In the DSIM framework, information and material spaces are explicitly separated, and the intelligent evolution of information that leads to if-then rules, bias, and actions is not part of the material space but develops naturally from the dual-space formulation (see Equation (1)). With this approach, information is allowed to grow dynamically, with individual agents independently evaluating the system. Some subset of rules and parameters must still be pre-defined, but individuals have their own unique view of the system. In practice, this can be applied to a wide range of models, but to examine how it functions this study will focus on a simple information asymmetry example.

## 3. Dual-Space Modeling of Information Asymmetry

The earliest research into asymmetric information influencing market evolution was established by George Akerloff (1970). In his work, the effects of asymmetric information are studied by introducing a range of qualities of products into a market and assuming that buyers are not informed about the quality of the product before they purchase it. To illustrate the concept, a used car market was described, where two qualities of cars were available (low and high, with fixed shares q and (1−q), respectively) and only the sellers knew the actual quality of a car prior to a sale. Buyers, not knowing the actual quality of cars for sale, would base their perceived value of a car on the average valuation of all cars in the market and be unwilling to pay more. Sellers would not want to sell high-quality cars at this lower price and would be driven from the market, resulting in only low-quality cars existing in the market. This adverse selection is a market inefficiency, and others have proposed methods to avoid its effects. Two prime examples include signaling in labor markets [12] and screening in insurance markets [20].

Of course, this is a simple example that does not consider many real-world variables that may affect the true value of a car (e.g., make, model, year, features, miles, etc.). Furthermore, it does not consider additional sources of information or sentiments (e.g., perceived trustworthiness of car salesman, friends’ opinions on different cars, comfort in/driving car, need for car, need for dealership to sell car, etc.) that may affect a buyer’s or seller’s value of the car. However, empirical investigations indicate that real markets exist with strong information asymmetry and no signaling [21,22]. To study the disconnect between early asymmetric information theory and real market behaviors, agent-based models have been employed [23,24,25]. These seek to establish the conditions which lead to market failure and to examine the evolution of these markets.

While these agent based modeling (ABM) use information measures, they are often static—as are the rules which govern their uses. This paper seeks to illustrate the benefits of DSIM-based AIM simulations when compared to traditional ABMs. To do so, a simple asymmetric information ABM will be studied, converted to AIM, and expanded into more complex AIM models that allow information to evolve in its own space. The traditional ABM used here is based on Wang, Li, and Liu [25].

### 3.1. Agent-Based Models of Simple Information Asymmetry

The information asymmetry ABM consists of 2N traders split equally into two classes (N buyers and N sellers) who trade a single product in a market. The quality of the product is heterogeneous and distributed randomly over a set range [a, b]. Each trader establishes a valuation of individual products according to the product’s quality and the trader’s preferences. The trading process is modeled by matching buyers and sellers randomly. Each pair (buyer and seller) will decide whether to complete transactions based on their own valuations. The total volume of sales and average price of products sold can be recorded after each timestep.

Seller k is selling a product of quality $q_{k}$ and is kept fixed throughout the simulation. This seller values the product linearly according to the quality ($r_{k}^{s}$ denotes the marginal cost of one additional unit of quality for seller k):

(2) $$V_{k}^{s}=r_{k}^{s}q_{k}$$

Similarly, buyer i values a good in the same way (with $r_{i}^{b}$ denoting the marginal value of one additional unit of quality for the buyer and with q denoting the quality):

(3) $$V_{i}^{b}=r_{i}^{b}q$$

However, the actual quality of a good will only be known by the buyer after a purchase, and thus information asymmetry exists. When matched with a seller, the buyer only has an expectation of the quality of the product, denoted by $q_{i}^{e}$. This is assumed to be the average value of products that have been traded, and we will limit this to an individual agent’s own experiences instead of the universal experiences of the market, similar to Wang et al. [25]. An initial value for $q_{i}^{e}$ is randomly assigned for each buyer, representing the pre-existing information of the agent.

Akerloff’s Lemon model assumed complete asymmetric information. However, Wang et al. [25] and others recognized that buyers will likely have some cognitive capability to determine the quality of a product. They denote this as parameter β, so that the quality of a product for a buyer can be written in the form:

(4) $$q=\beta q_{k}+\left(1-\beta \right)q_{i}^{e}$$

β itself can range from 0 to 1. β=0 indicates that the buyer is unable to assess the true quality of a product, while β=1 indicates that the buyer has complete information and identifies the exact quality.

In each timestep of the simulation, each seller takes one unit of their product to the market, and each buyer comes to the market with their own expectations of the quality of the good they plan to buy. The buyers and sellers are randomly matched, so that seller k meets buyer i. For each pair, a transaction will only occur if the valuation of the buyer is at least as great as the valuation of the seller:

(5) $$V_{i}^{b}\geq V_{k}^{s}$$

Combining the above, the condition for the sale becomes:

(6) $$r_{i}^{b}\left[\beta q_{k}+\left(1-\beta \right)q_{i}^{e}\right]\geq r_{k}^{s}q_{k}$$

This, in turn, can be simplified by letting all buyers have the same preference and all sellers operate at the same mode, so that the subscripts of r can be dropped. This leaves the ratio $\lambda =r^{b}/r^{s}$ as the ratio of valuation between buyers and sellers of the same product with complete information.

(7) $$\lambda \beta +\lambda \left(1-\beta \right)\frac{q_{i}^{e}}{q_{k}}\geq 1$$

Updating this model to AIM requires a recognition of which variables are information based and which are material based, as well as what rules exist. In this case, the only material space variable is the actual quality of a product. The valuations and ability to determine the actual quality (r and β, respectively) reside in the information space of each buyer agent. Market efficiency is defined here as the percent of possible transactions that actually occur.

Three rules exist to: (1) update the expected quality of products for an agent, $q_{i}^{e}$, (2) determine the perceived quality of a product, q, and (3) determine if a transaction will occur, $V_{i}^{b}\geq V_{k}^{s}$. The first exists purely in the information space of buyers, the second interprets the material space into an information space variable by the buyers, and the third compares the presented information space of the buyer and the seller without revealing the actual components of their information space.

**Figure 2:**
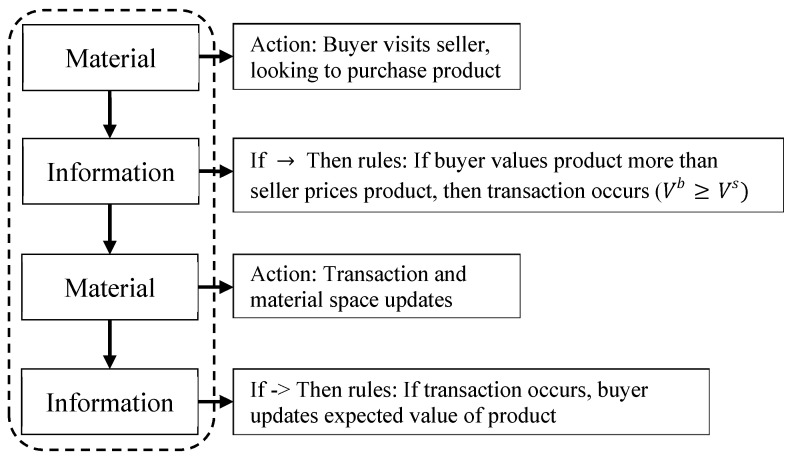
The ABM implementation of asymmetric information model follows a sequential format. This alternates between actions which alter the material variables and if->then rules working with information variables and determining what material actions will occur. In ABM, there is no actual distinction between material and information.

**Figure 3:**
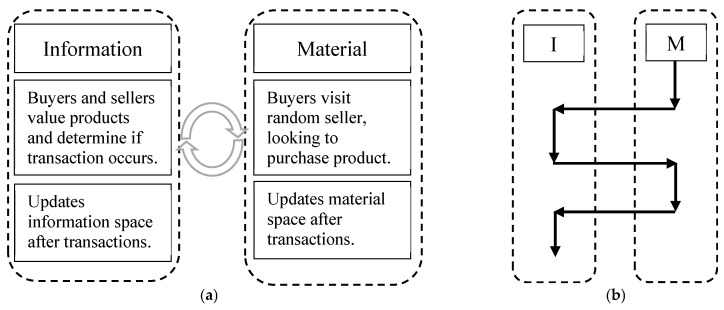
The AIM implementation of asymmetric information model follows a parallel format. (**a**) generalized AIM treatment; (**b**) for the simple asymmetric information models, AIM can be viewed as sequential step-wise processes equivalent to the ABM.

Functionally, the ABM and AIM versions of the model act in the same way and produce the same results (i.e., the average value of products sold and market efficiency, shown below). The same actions and rules occur in each case. However, the separation into the dual-space allows AIM models the flexibility to change and expand the rules in the information space, in addition to providing a cleaner, clearer description of what and how the interactions occur. Based on the conceptual diagram shown in Figure 1, the implementation of both the traditional ABM and the dual-space AIM are illustrated in Figure 2 and Figure 3 to help differentiate between the two methods.

The usual ABM approach to modeling information asymmetry is diagramed in Figure 2. Typically, the material space and information space are not separated and are mixed in a single body, so that the process occurs in a sequential fashion going through material and information entities. The steps are separated by actions (occurring primarily among material aspects) and rules (occurring primarily among information aspects) that determine which actions will occur. The distinction between material actions and information rules are shown here to be helpful when compared to AIM. However, there is no separation between the material and information spaces in ABM, and both are treated equally. Furthermore, minimal processing occurs over the information variables (simply calculating and comparing values of the product), and minimal evolution occurs (updating the perceived value of the product).

In contrast to the traditional ABM, AIM allows for the parallel processing of the material and information spaces (Figure 3a). In the simple asymmetric information model, the two modeling methods function similarly. In fact, the original ABM setup can be obtained by looking at it as a step function version of AIM, where the simulation switches between the material space and information space at each step (Figure 3b). The key difference in the simplistic model is a recognition of the distinction between material and information, which allows for more flexible modifications to the model.

**Figure 4:**
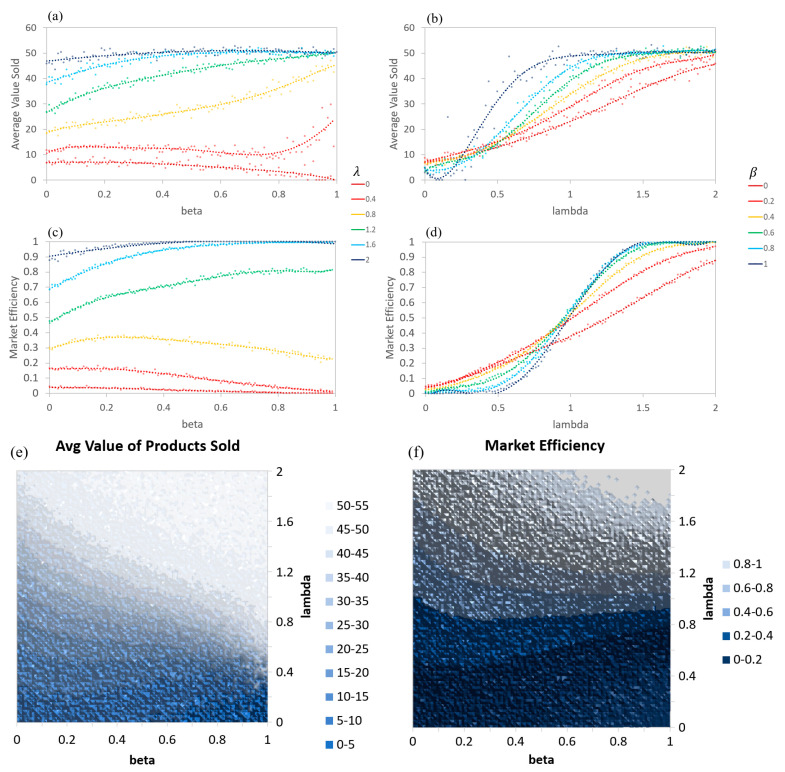
ABM and AIM produce nearly identical results in the simple asymmetric information models. Here, β is a constant for each agent, forming a gaussian distribution around the reported value (with limits of 0 and 1). (**a**) Average value of products sold as a function of β for specific λs, (**b**) Average value of products sold as a function of λ for specific βs, (**c**) Market efficiency as a function of β for specific λs, (**d**) Market efficiency as function of λ for specific βs, (**e**) Average value of products’ sold contour, and (**f**) Market efficiency contour.

Both the ABM and AIM versions of the simple asymmetric information model produce nearly identical results (up to the random variance). As such, the graphs in Figure 4 will only show the AIM results. In this example, β is set to a constant for each agent, forming a Gaussian distribution with a mean of the reported β and limited to a minimum of 0 and maximum of 1. λs are ratios of the buyer over seller valuations of the products that are set to distribute from 0 to 2. Equal numbers of buying and selling agents were paired at each timestep, and the values of a random quality of product were compared to determine if a transaction would occur.

Generally, low levels of information asymmetry (large β) and high levels of relative valuation (large λ) result in more products sold and at higher prices (Figure 4a,b,e). Higher levels of information asymmetry (low β) and lower levels of relative valuation (low λ) lead to weaker, less efficient markets (fewer products sold and at lower prices) (Figure 4c,d,f). Additionally, a larger β also results in the graphs of the average price and number of products sold showing more sigmoidal shapes when λ is varied (Figure 4b,d). For a system of buyers all having β=1, this would be a step-function, as λ transitions from less than 1 to greater than 1. Figure 4e,f demonstrates the multi-variate behavior of the market in 2D density plots.

Agents with a weak ability to determine the actual quality of a product will rely more on their expected quality of a product based on previous transactions. As such, it is difficult for them to think a product is of a higher quality than the average quality of products that they have encountered, leading to an expectation that products are of a lesser quality than they are and to fewer transactions in general. This relates directly to the Market for Lemons model, where higher priced quality products are essentially priced out of the market and where mainly lower priced poor-quality products sell. However, the complete market collapse is not seen. How $q^{e}$ is calculated for each agent may be responsible for the difference. Here, agents only have knowledge of product qualities from their own transactions, while in the original Akerloff model the agents knew the average quality of all of the products.

### 3.2. Agent and Information Models of Information Asymmetry

Diverging from traditional ABM requires an increased emphasis on the information and material space separations. Where AIM begins to shine as more than just an alternative way of thinking about and setting up an ABM is in more complex models. For example, the next model includes the ability for β (an information measure) to improve through buyers interacting with each other (either within groups or with all other buyers; however, the exact means of growth can vary between real populations and within simulations). This is more reminiscent of a real ability where, over time, a person may be able to improve their knowledge and cognitive ability to determine the quality of goods, either by learning from others (as shown in this model) or by an accumulation of experiences. This is easily accomplished in AIM by allowing β to change between transactions and by assigning groups for buyers to share information within.

In an ABM, β is treated as just another variable that is associated with agents. The simplest cases use a constant β, which is the same for every agent. Allowing β to be assigned independently for each agent is common, but the evolution of β is rarely studied. The agents change their expected value of a product based on previously seen products, but never improve their ability to determine the true value. This is accomplished simply and naturally in AIM by defining β as an information space variable, which has its own dynamics and independent evolution.

To more clearly understand the dynamics of the model, the number of sellers is reduced (five in this case), each of which will mark up the price of their products by a constant rate relative to their quality. This is equivalent to giving each seller a set $r^{b}>1$. The evolution of the sellers’ material space can then be examined with respect to the initial conditions in the system’s information space, such as by controlling the initial β or its evolution.

When β is allowed to grow through the sharing of information with other agents within groups, the buyers are better able to determine the actual quality of products. Initially, buyer agents are assigned to random groups, in which buyers learn from each other, with the weaker β members learning the most, so that all agents slowly approach the β of the strongest member. The strongest member can learn from the weaker members, but at a much-reduced rate. This is represented mathematically with Equation (8) for the case of buyer b belonging to group g, which has $N_{g}$ members in the group and a learning efficiency $\eta_{b}$:

(8) $$\beta_{b}\left(t+\Delta t\right)=\beta_{b}\left(t\right)+\eta_{b}\left(1-\beta_{b}\left(t\right)\right)\left(\underset{i\in g,\;i\neq b}{\sum }\frac{\beta_{i}\left(t\right)}{N_{g}}\right)$$

This leads to clusters of agents with similar βs. However, with a large number of agents in a simulation, the clusters themselves combine to form a normal distribution of β.

**Figure 5:**
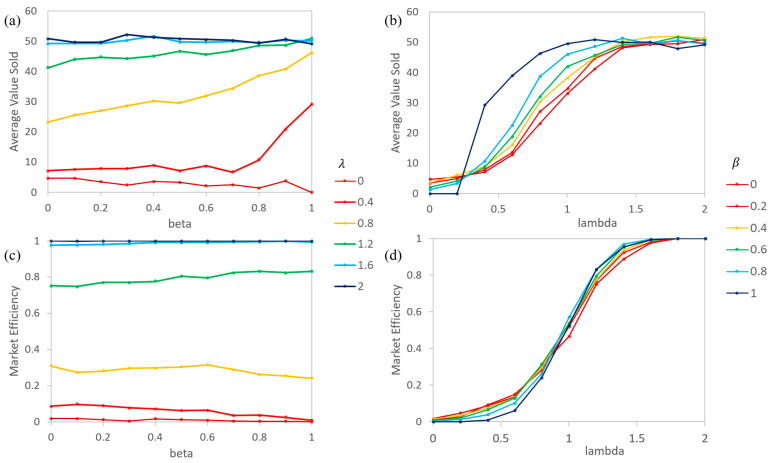
AIM results for the simulation with β growth by sharing with other agents within groups after the simulation reaches steady-state. (**a**) Average value of products sold as a function of β for specific λs, (**b**) Average value of products sold as a function of λ for specific βs, (**c**) Market efficiency as a function of β for specific λs, and (**d**) Market efficiency as a function of λ for specific βs. Compared to the static β case, more agents improved their βs from sharing and were able to better determine the true quality of products.

Figure 5 demonstrates the market efficiency (Figure 5c,d) and average price of products sold (Figure 5a,b). Compared to the simple model, the λ varying graphs show that each line shifts closer to the higher β lines, as expected when β increases during the simulation (compare Figure 5b,d to Figure 4b,d). Shifts are seen in the β varying graphs where the lines of the initial λ are more condensed at a high and low efficiency and at the price levels (similar to the simple model at a higher β, compare Figure 5a,c to Figure 4a,c). Generally, this would suggest that in any market where agents are able to improve their ability to determine information, high asymmetry (a low β) results would be transitory at best. Mid-to-low asymmetry (a medium to large β) would then have to be modeled to understand long term behaviors.

**Figure 6:**
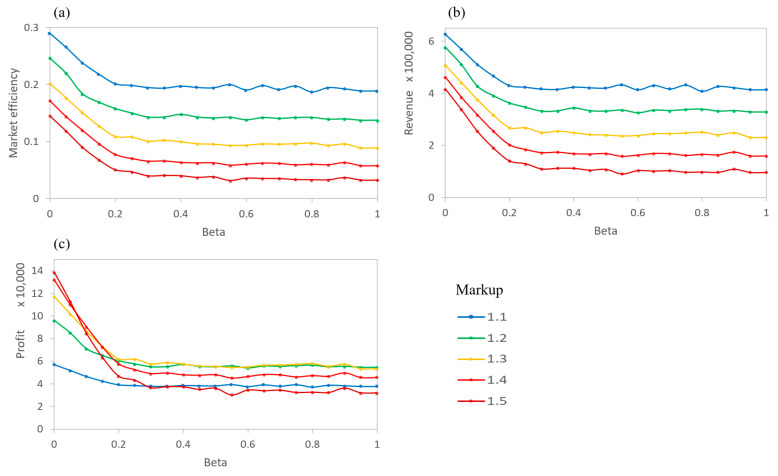
AIM simulation of 5 sellers, each of which marks up the price of a product by a different rate. All are averages over the final 10 timesteps. (**a**) Market Efficiency for each seller, (**b**) Revenue for each seller, and (**c**) Profit for each seller.

When sellers mark up their products at different rates, the total revenue, market efficiency, and profit for each seller become interesting to examine (shown in Figure 6). Profit is defined as the price sold minus the actual value of a product. No penalties are given for not selling (or not buying) a product during a timestep, but buyers only visit one agent and see one product per timestep. As expected, the market efficiency and the total revenue decreases as the markup rate increases (Figure 6a,b). That is, few buyer agents are willing to pay significantly more than the actual value of a product (defined by the actual quality of the product). This holds for all levels of information asymmetry. At mid and high levels of asymmetry (β>0.2), the total revenue and number of products sold are nearly constant for individual sellers. This suggests that for the buyers, a moderate level of β represents a sufficient ability in determining the relative prices of products. However, at a high asymmetry (a low β) the number of products sold and the revenue increase greatly. Sellers are able to sell more products, especially for those with higher markup rates, by taking advantage of buyers’ inability to determine the true quality of the products.

The plot for profits then shows the interaction between the number of products sold and total revenue (Figure 6c). Instead of the tiered lines, as in the volume and revenue graphs, the profits intersect, so that different levels of markups are optimal for different levels of β of the buyers. High asymmetry buyers lead to high levels of profit for the sellers with high markups. However, the profits for high markup sellers sharply decrease with a weaker asymmetry. For β>0.2, the markup level for the greatest profits is a midrange markup ($r_{k}^{s}=1.2\sim 1.3$).

**Figure 7:**
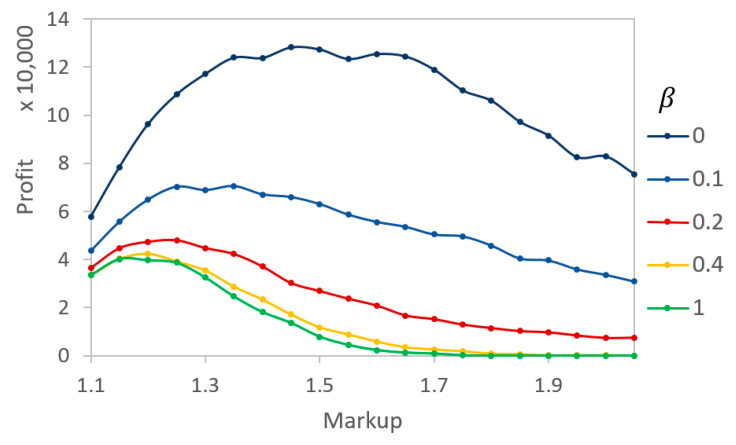
Relative profit for different βs as a rate of markup changes. At each β, there exists an optimal markup value for the seller to make optimal profits.

To further examine the profits, simulations were run for varying levels of markup at distinct β values. Figure 7 demonstrates the optimal markup values for each of the given βs. For a low β, the markup is high because agents are unable to reasonably determine the actual value of a product. For larger βs (β~0.2), the optimal markup value decreases to the 20% range. This is common in real markets where profit optimization is a multivariable function of the price, volume, and costs. The results of the situation show that it would be beneficial for sellers to understand their buyers’ level of ability to determine the actual quality (i.e., the level of information asymmetry) before determining their selling prices.

Note that while profits decrease with higher markups, they do not become extremely small in the simulated range. However, this model neglects any additional costs that may lower the profits and calculates the profit as the difference between the sales revenue and the always smaller cost of the product. In other words, every transaction will yield a profit.

### 3.3. More Complex Models of Information Asymmetry

In this section, in order to further exemplify extensions using the AIM framework, more complexity will be added to the information space variables by allowing buyers and sellers to adjust their prices based on individual needs. The sellers will need to average a profit of at least P every timestep in order to cover unspecified costs (wages, rent, etc.). Each timestep, their need, $n^{S}$, will increase by P and decrease by the actual profit from their successful transactions ($V^{S^{\prime }}-Q$). The price they attempt to sell a product for is now $V^{S^{\prime }}$ and is moderated through a sigmoid function, as shown below (with $\alpha^{S}$ a constant describing the range of the sigmoid):

(9) $$V^{S^{\prime }}=\frac{V^{S}}{\left(1.0+\alpha^{S}\left(\frac{1}{1+e^{-\frac{n}{P}}}-0.5\right)\right)}$$

This leads to the sellers being able to increase their price for more profit margins when products are selling well ($n^{S}<0$) and decrease their price to promote sales when they need to sell more ($n^{S}>0$). At a high magnitude $n^{S}$, the sigmoid saturates, so that the range of $V^{S^{\prime }}$ is limited:

(10) $$\frac{V^{S}}{1-\frac{\alpha^{S}}{2}}\geq V^{S^{\prime }}\geq \;\frac{V^{S}}{1+\frac{\alpha^{S}}{2}}$$

The need determines how much or little to mark up the price of a product based on the sigmoidal function shown. The sigmoid, or similar function, is necessary for establishing limits on the prices of the products. In other words, it is a mechanism of the seller to ensure that products will be priced above the cost (on the low end) and within a reasonable range (on the high end).

Buyers, on the other hand, need to, on average, purchase one product every T timesteps. Similar to the sellers, the buyers’ needs $n^{B}$ will increase by 1/T every timestep and decrease by 1 for every product they purchase. The max price they will buy a product for during a timestep is $V^{B^{\prime }}$ and is moderated through a sigmoid function, as shown below (with $\alpha^{B}$ a constant describing the range of the sigmoid):

(11) $$V^{B^{\prime }}=V^{B}\times \left(1.0+\alpha^{B}\left(\frac{1}{1+e^{-\left(n^{B}\;T\right)}}-0.5\right)\right)$$

This leads to the buyer being able to pay more when they have not bought enough yet ($n^{B}>0$) and only being willing to pay less when they have more than enough purchases at the time ($n^{B}<0$). At a high magnitude $n^{B}$, the sigmoid saturates, so that the range of $V^{B^{\prime }}$ is limited:

(12) $$V^{B}\left(1-\frac{\alpha^{B}}{2}\right)\leq V^{B^{\prime }}\leq V^{B}\left(1+\frac{\alpha^{B}}{2}\right)$$

**Figure 8:**
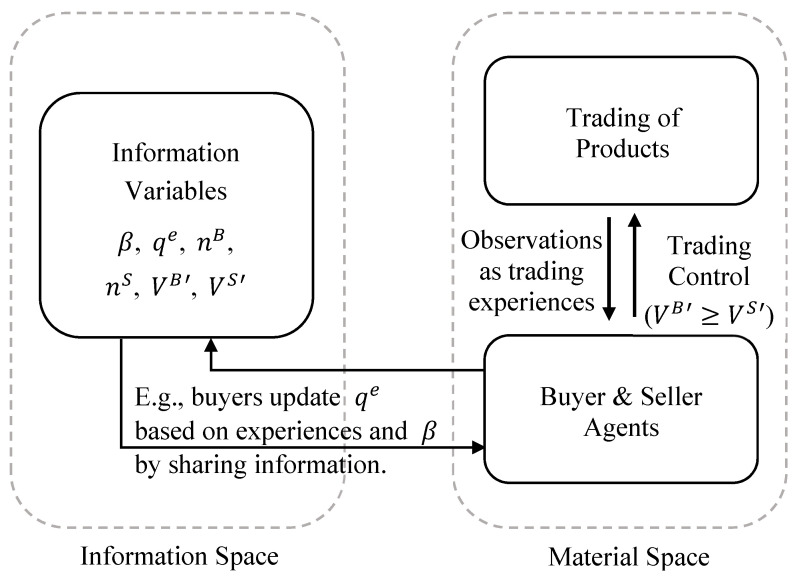
A DSIM representation of a more complex market model of information asymmetry. Here, sellers will mark up the price of their products at different rates and buyers’ ability to determine the quality of a product will improve based on the sharing of information with other buyers. Additionally, the rate of markups and the price that buyers are willing to pay will adjust based on individual buyers’ and sellers’ needs.

The entire process is summarized in Figure 8, which provides a specific example of the conceptual diagram shown in Figure 1. In this model, the material space processes are similar to the simple models discussed previously. When conditions are met, trades of materials will happen. On the other hand, the information space is more complex than that of the previous models. It has a number of information variables, including buyers’ abilities to determine product quality, buyers’ estimations of product quality, and the needs of buyers and sellers. These are allowed to evolve among themselves through intelligence-based processes such as improving buyers’ abilities through the accumulation of trading experiences and sharing from other buyers. In this way, the information variables can establish intelligence-based evolution dynamics within their own space, which are mostly independent of the material space processes. The results of the information processes can then be applied by the agents during decision making to impact material-based interactions such as the trading of products.

**Figure 9:**
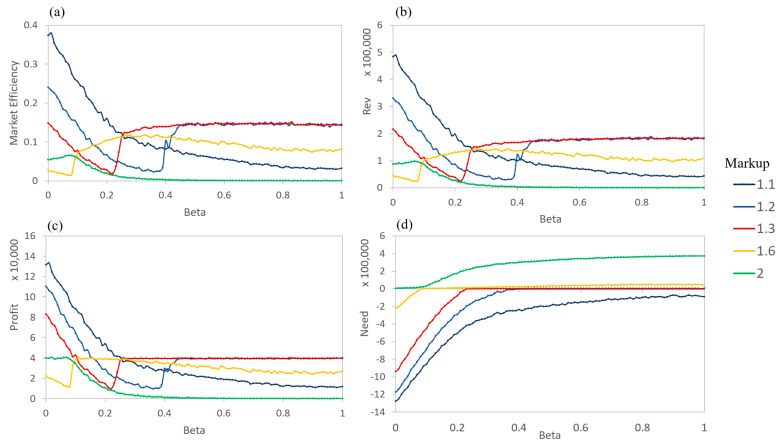
Seller performance based on β and the degree of markup. (**a**) Market efficiency (over last 10 timesteps), (**b**) Revenue (over last 10 timesteps), (**c**) Profit (over last 10 timesteps), and (**d**) Need (cumulative over entire simulation).

**Figure 10:**
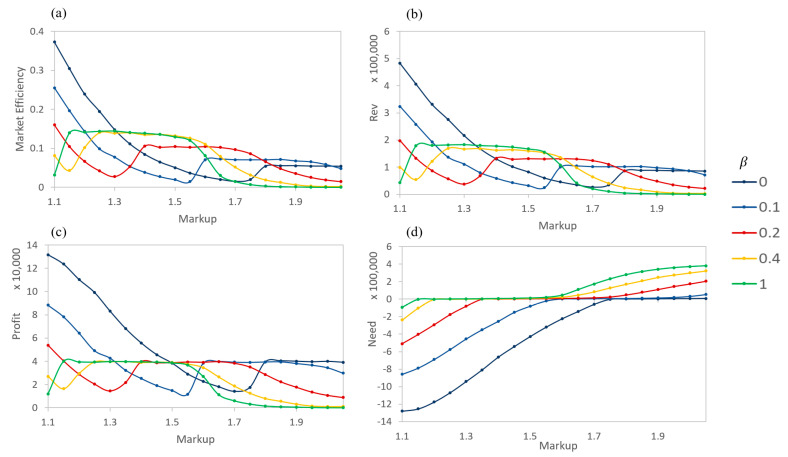
Seller performance based on degree of markup and β. (**a**) Market efficiency (over last 10 timesteps), (**b**) Revenue (over last 10 timesteps), (**c**) Profit (over last 10 timesteps), (**d**) Need (cumulative over entire simulation).

The results of the simulation are inspected in terms of the performance of the sellers, whose prices adjusts based on their profit needs. Generally speaking, the sellers’ performance can be classified into four regions of β and markup (Figure 9 and Figure 10). (1) decreasing at a low β and low markup, (2) discontinuous jumps in performance occurring from a low markup to a high markup for buyers from a high β to a low β, (3) continuous increase or near constant performance at a medium β and a medium markup, and (4) slow decrease at a high β and a high markup. Not every seller will cover all four regions due to constraints on β and the markup. The discontinuities occur where need transitions from negative to positive. Note that “need” is calculated with the cumulative deficit (positive) or excess (negative) in profit over the life of the simulation, so that the seller would operate in a way that is long-term sustainable. Meanwhile, the market efficiency, revenue, and profit are moving averages that are calculated over the last 10 timesteps of the simulation in order to demonstrate short-term marketing strategies and performances. Obviously, these modeling specifics are open for exploration depending on the contexts and goals of a study.

**Figure 11:**
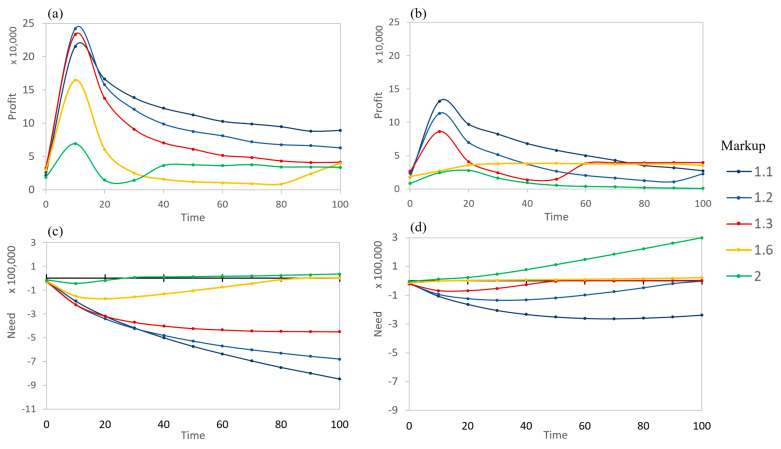
Profit (averaged over 10 timesteps) and need (cumulative) time evolution for various markup amounts using populations with initial average βs. (**a**) Profit for β=0.1, and (**b**) Profit for β=0.4, (**c**) Need for β=0.1, and (**d**) Need for β=0.4. Generally, the profits are increased in magnitude as β lessens.

Based on the conditions of this study, it can be observed that high βs often result in a stable (with respect to β) market behavior (Figure 9). When buyers can nearly determine the exact value of a product, the system settles into balancing the needs of the buyers and sellers. When balanced, the change in a seller’s need over a period is near zero as the incoming profit matches the needed profit. However, not every seller is able to reach a balance with their need. Those with larger markups are unwilling to lower their prices to a level that will easily sell due to range limitations imposed by the sigmoidal value function. At this point, the trading for these sellers basically stops (Figure 11). Those with minimal markups can profit the most early in the simulation and have a minimal need later (Figure 11). This is due to the fact that a random buyer visiting the low-markup sellers is more likely to complete a purchase than a random buyer visiting a high-markup seller is; additionally, many buyers fulfill their need early during the simulation, and only a subset of buyers have a significant positive need later.

At low βs, the results are more varied. Larger markup products are still more difficult to sell but can achieve needed profits over certain ranges of low β (Figure 9 and Figure 10). Still, they are generally outperformed by lower markups. Within the low-to-mid β range, there exist discontinuities where the performance drastically increases. These occur at β levels where sellers transition from being able to make the needed profits to where they are unable to achieve the profit (over the course of the entire simulation), at which point their needs go from negative to positive (Figure 9). At these discontinuous transitions, the sellers have lowered their prices to critical points that allow them to sell more products for more overall profits. Relating to reality, these transitions reflect features of marketing promotions, which lower prices to boost product sales and overall profits.

Similarly, Figure 10 shows the seller performance as the markup varies for specific βs. These reveal a similar behavior as in Figure 9, but allow for additional comparisons. In particular, for each measure, the different βs each result in the same shape as other βs. The transition points of the seller performance with buyers having higher levels of asymmetry are shifted right, towards higher markups and vice versa. The need graph then reveals regions of markup where the sellers will be very profitable (negative portion), regions where the sellers will only make the required profit overall (zero/flat portion), and regions where the sellers will lose money over the course of the simulation (positive portion). This places limits on possible long-term profits based on the parameters used in this simulation.

Figure 9 and Figure 10 show the stable states of the markets. It would be useful to inspect how markets evolve and arrive at their long-term stability. Figure 11 examines the time evolution of the seller markets based on differing markups and betas. All markup levels see a decrease in profits as time increases, caused by the needs of both the sellers and buyers being satisfied. This lowered profit region occurs when the need is becoming negative (total profits are good) yet the price of the product is high for buyers. However, some of the sellers see an increase in profits shortly after this, depending on the degree of information asymmetry that is present. The rise occurs when the need increases to near zero.

The need corresponds to the overall performance of a seller (difference between the total amount needed and the total profits). All sellers perform better early, although high markup sellers may still never achieve the needed amount if β is high (i.e., green line in Figure 11a). Generally, lower markups result in an improved overall performance for all times, even if the short-term profits are lower. During early timesteps, some slightly raised markups can overtake lower markups (red vs. blue) at a low β.

## 4. Discussion

Overall, AIM has shown that the separation of the material and information spaces leads to new ways of thinking about ABM and new approaches to modeling in general, through the careful consideration of how and where interactions take place between the dual spaces, and which parts of a simulation belong to each space. A key understanding of the differentiation between the two spaces is recognizing that the material space is conserved, exchanging or using goods within the material space so that the space itself is bounded. The information space, on the other hand, is based on intelligent processes, and it is unbounded and allowed to grow. A typical algorithm to handle the growing information space in order to produce a usable probability is the use of normalization over a desired range of information variable changes.

Through this paper, AIM has been shown to reproduce the results of a traditional ABM asymmetric information model, as well as to enhance it through the evolution in the information space. These evolutions allow for more real-world-like intelligent behavior, so that agents can learn and share with one another, even without a direct effect on the material space. This leads to agents having an expanded understanding of, in this case, how to accurately determine the quality of a product and the actual average quality of available products.

More complex AIMs begin to further differentiate AIM from ABM by allowing a large range of varied behaviors to occur in the different spaces. Introducing “need” to both the buyers and sellers required each to consider their individual material and information spaces in order to determine their actions. What resulted was a complex set of behaviors leading to sellers adjusting their prices so as to best increase sales to the required profit levels. As a multi-dimensional model, this may easily be expanded to further measures of needs or products in order to form a more complete and realistic simulation.

Beyond the information asymmetry models discussed here, AIM can be applied to wide ranging systems with intelligent agents, including other economic systems, social dynamics, learning, etc. Additionally, AIM can be used as a tool to aid in the learning of complex systems when analytic solutions are not readily available or understood. Through the DSIM framework, one can organize an agent-based simulation in a simple way, in order to model how the interactions between information and material spaces via intelligent agents lead to complex emergent behaviors that are sometimes unexpected.
